# Feasibility of Novel Software-Based Perfusion Indicators for the Ileal J-Pouch—On the Path towards Objective and Quantifiable Intraoperative Perfusion Assessment with Indocyanine Green Near-Infrared Fluorescence

**DOI:** 10.3390/life12081144

**Published:** 2022-07-28

**Authors:** Leonard A. Lobbes, Richelle J. M. Hoveling, Susanne Berns, Leonard R. Schmidt, Rahel M. Strobel, Christian Schineis, Johannes C. Lauscher, Katharina Beyer, Benjamin Weixler

**Affiliations:** 1 Department of General and Visceral Surgery, Charité—Universitätsmedizin Berlin, Corporate Member of Freie Universität Berlin and Humboldt-Universität zu Berlin, Hindenburgdamm 30, 12203 Berlin, Germany; susanne.berns@charite.de (S.B.); leonard.schmidt@charite.de (L.R.S.); rahel.strobel@charite.de (R.M.S.); christian.schineis@charite.de (C.S.); johannes.lauscher@charite.de (J.C.L.); katharina.beyer2@charite.de (K.B.); benjamin.weixler@charite.de (B.W.); 2 Quest Medical Imaging, 1775 PW Middenmeer, The Netherlands; richelle.hoveling@quest-innovations.com

**Keywords:** J-pouch, IPAA, anastomotic leak, ulcerative colitis, restorative proctocolectomy, perfusion assessment, indocyanine green, ICG, near-infrared fluorescence, pixel-to-pixel perfusion mapping, perfusion-time curve

## Abstract

Background: In restorative proctocolectomy with ileal J-pouch, perfusion assessment is vital to prevent complications such as anastomotic leak (AL). Indocyanine green near-infrared fluorescence (ICG-NIRF) is gaining popularity, while its interpretation and relevance remain subjective. This study aimed to evaluate a standardized ICG-NIRF imaging protocol combined with a novel, software-based assessment to detect areas of impaired perfusion and a possible correlation with AL of the pouch. Methods: In this prospective study, patients undergoing ileal J-pouch for ulcerative colitis at an inflammatory bowel disease (IBD) referral center were included. Intraoperatively, strictly standardized ICG-NIRF visualization was performed and video-recorded. Postoperatively, a specific software was utilized to determine the change in fluorescence intensity per second (i/s) for systematic regions of interest, generating perfusion-time curves and a pixel-to-pixel map. These were analysed in detail and correlated with clinical outcome (primary end point: AL within 30 days; clearly defined and screened for by pouchoscopy). Results: Four out of 18 included patients developed AL of the ileal pouch-anal anastomosis (IPAA). In the AL group, the perfusion curves on the area adjacent to the IPAA (pouch apex) displayed considerably lower ingress/inflow (median = 1.7; range = 8.5; interquartile-range = 3.8 i/s) and egress/outflow (median = −0.1; range = 0.7; interquartile-range = 0.5 i/s) values than in the non-AL group (ingress: median = 4.3; range = 10.3; interquartile-range = 4.0 i/s); egress: median = (−1.1); range = 3.9; interquartile range = 1.0 i/s). This was confirmed by further novel parameters of pouch perfusion (maximum ingress; maximum egress) and pixel-to-pixel analysis. Conclusions: This study presents the feasibility of a novel methodology to precisely assess pouch perfusion with ICG-NIRF, identifying comparable, quantifiable, and objective parameters to potentially detect perfusion-associated complications in surgery in real-time.

## 1. Introduction

Restorative proctocolectomy (RPC) with the construction of an ileal J-pouch and ileal pouch-anal anastomosis (IPAA) has been established as the surgical procedure of choice for patients with medically refractory ulcerative colitis (UC) or familial adenomatous polyposis (FAP) and selected patients with Crohn’s colitis [1,2,3]. A two- or three-stage approach is usually chosen, depending on the individual patient’s condition and risk factors [4,5]. The J-pouch and IPAA are presently usually fashioned by mechanical stapling, although a hand-sewn anastomosis can be required, especially after mucosectomy [6]. While RPC generally improves quality of life with favorable long-term functional outcomes, complications such as anastomotic leak (AL) of the pouch with pelvic sepsis, pouch necrosis, strictures, fistulas and sinuses as well as pouchitis can severely affect patient outcome [6,7,8,9,10,11]. Especially early complications including AL and pelvic sepsis increase the risk of pouch failure and long-term morbidity [11,12,13]. AL of the pouch has a reported incidence of 5–20%, while associated local septic complications have a reported incidence of 7–36% [14,15,16,17].

The risk of AL is increased by an impaired blood flow to the pouch and IPAA [18,19]. Consequently, performing an accurate perfusion assessment and a tension-free anastomosis during pouch formation are crucial. Currently, fluorescence-guided imaging techniques such as indocyanine green-derived near-infrared fluorescence (ICG-NIRF) are increasingly being used for intraoperative intestinal perfusion assessment, additionally to conventional parameters (such as intestinal colour, bleeding from transection sites, pulse of vascular arcades) [20,21,22,23,24,25]. ICG-NIRF allows the surgeon to visualise bowel perfusion in real-time and may have potential to reduce the incidence of AL [22,25,26]. Nevertheless, its interpretation is still subjective, predominantly consisting of an individual real-time assessment of a fluorescence or overlay image. As with conventional clinical parameters of perfusion, the validity of ICG-NIRF perfusion visualization is highly dependent on the individual experience and degree of specialization of the surgeon [27]. To date, the accuracy and sensitivity for the detection not only of an absolute lack of perfusion, but rather of venous congestion and other degrees of (micro-) perfusion impairment and the AL-associated perfusion indicators of ICG-NIRF are unknown. Approaches for quantifying ICG-NIRF for bowel perfusion assessment have been described occasionally but have not been implemented or validated mechanistically [23,28,29,30]. A quantitative and objective approach of ICG-NIRF perfusion assessment has not been described for pouch surgery. Additionally, in the existing literature, the intraoperative ICG-NIRF measuring environment is not standardized, resulting in differing or variable ICG doses, measuring time points, surrounding light, and distances of the detection device and light source to the operating field. This makes a comparison of individual visualizations and related scientific findings difficult, if not impossible. 

This study aimed to evaluate a systematic approach of intraoperative ICG-NIRF imaging (with standardized measuring time points, dosage and distance) combined with a software-based perfusion assessment for the detection of areas of impaired perfusion by correlation with postoperative outcome including AL of the pouch. We hypothesized that a dynamic depiction of fluorescence intensity over time would result in novel, quantifiable and objective indicators of pouch perfusion.

## 2. Materials and Methods

This prospective cohort study (NCT04695184) was conducted from February 2019 to December 2020 at the Department of General and Visceral Surgery, Charité University Medicine Berlin, Campus Benjamin Franklin, a tertiary referral center for colorectal and inflammatory bowel disease (IBD) surgery. Study approval was given by the Charité University Hospital Ethics Committee (No. EA4/116/19; Date of approval: 24 January 2019) and all data were reported in conformity with the STROBE guidelines [31]. 

Patients receiving RPC with an ileal J-pouch in either a two- or three-stage approach were included. In the two-stage approach, patients underwent proctocolectomy simultaneously with ileal J-pouch and protective ileostomy, followed by ileostomy reversal. Patients undergoing the three-stage approach received subtotal colectomy with end ileostomy in stage one, completion proctectomy with ileal J-pouch formation and loop ileostomy in stage two, and ileostomy reversal in stage three.

Study inclusion required a diagnosis of medically refractory UC, indeterminate colitis (IC), Crohn´s colitis or familial adenomatous polyposis (FAP), age ≥ 18 years and an American Society of Anesthesiologists (ASA) physical status ≤ 3. Patients were excluded if they displayed a hypersensitivity to ICG or sodium iodide, iodine allergy, hyperthyroidism, thyroid nodules, a previously poorly tolerated injection of ICG, pregnancy or breastfeeding, a coexisting malignancy or liver dysfunction (with a Model End Score Liver Disease (MELD) score > 10).

Informed consent for study participation was received at least 24 h prior to surgery.

### 2.1. Surgical Technique

Following the standard surgical operating procedure of the tertiary IBD referral center, laparoscopic preparation was followed by fashioning of the J-pouch to a length of 14–16 centimetres (cm) by linear single-stapling. It was then ensured that the fashioned J-pouch had sufficient mobility to extracorporeally reach down to the symphysis pubis without tension, to enable tension-free IPAA. IPAA was achieved by circular mechanical double-stapling using an end-to-end anastomosis (EEA) 29 millimeter (mm) stapler. In cases where mucosectomy was required due to risk of malignancy, single-layer hand-sewn anastomosis was performed after completed mucosectomy. In cases where laparoscopic preparation was considered a risk for patient safety, open surgery was conducted.

### 2.2. Intraoperative ICG-NIRF Perfusion Visualization

**Figure 1:**
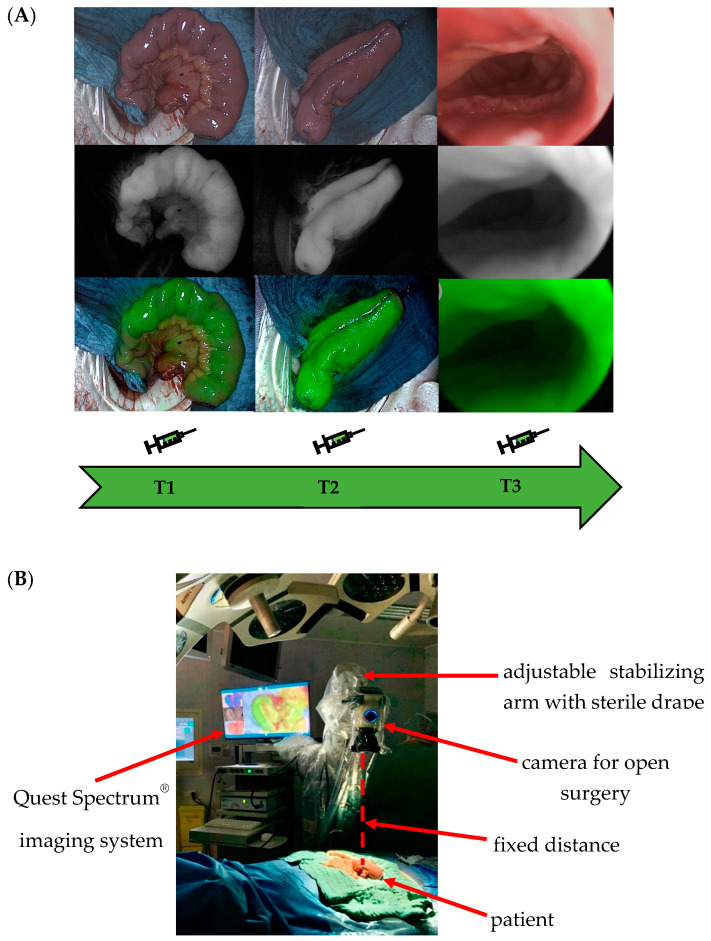
(**A**) Visualization at three predefined time points during ileal J-pouch formation. Time point T1: prior to J-pouch fashioning. Time point T2: after formation by side-to-side stapled anastomosis. Time point T3: visualization of the completed ileal pouch-anal anastomosis (IPAA). (**B**) Standardization of the imaging environment. The Quest Spectrum^®^ system was secured in position with a stabilizing arm to ensure a fixed distance to the operating site and adjusted for focus and field of view. At each time point, the effect of surrounding light was minimized by turning off the operating room and surgical lights. Each visualization was video recorded for 120 s to enable postoperative analysis. Patient baseline, intraoperative clinical and technical imaging environment data were documented, as well as the timing between the measurements. This set-up was designed to achieve a standardized imaging environment to allow comparability between individual measurements.

During ileal J-pouch formation, intraoperative real-time perfusion visualization was performed with the Quest Spectrum^®^ Fluorescence Imaging Platform (Quest Medical Imaging, Middenmeer, The Netherlands) at three consecutive, predefined measuring time points (Figure 1A). ICG (VerDye, Diagnostic Green GmbH, Aschheim, Germany, 25 mg) was dissolved in 5 mL sterile water to yield a 5 mg/mL concentration and administered intravenously as a bolus of 1 mL (5 mg) at each time point.

At time point 1 (T1), prior to J-pouch formation, the terminal ileum was visualized to ensure adequate baseline perfusion. At time point 2 (T2), the J-pouch was visualized immediately after its formation by side-to-side stapled anastomosis. At time point 3 (T3), after trans-anal circular stapling, the completed ileal pouch-anal anastomosis (IPAA) was visualized. At time points 1 and 2, the Quest Spectrum^®^ system was equipped with the ring light camera for open surgery, which was secured in position with a stabilizing arm to ensure a fixed distance to the operating site and adjusted for focus and field of view (Figure 1B). At time point 3, the IPAA was visualized at 360 degrees by inserting the Quest Spectrum^®^ laparoscope anally via a covered 12 mm laparoscopy port (Versaport™ Bladeless Optical Trocar, Medtronic, Minneapolis, MN, USA), protecting the IPAA while allowing manual air insufflation.

At each time point, the effect of surrounding light was minimized by turning off the operating room and surgical lights. Each visualization was video recorded for 120 s to enable postoperative analysis. Patient baseline, intraoperative clinical and technical imaging environment data were documented, as well as the timing between the measurements.

### 2.3. Postoperative ICG-NIRF Perfusion Rate Assessment

**Figure 2:**
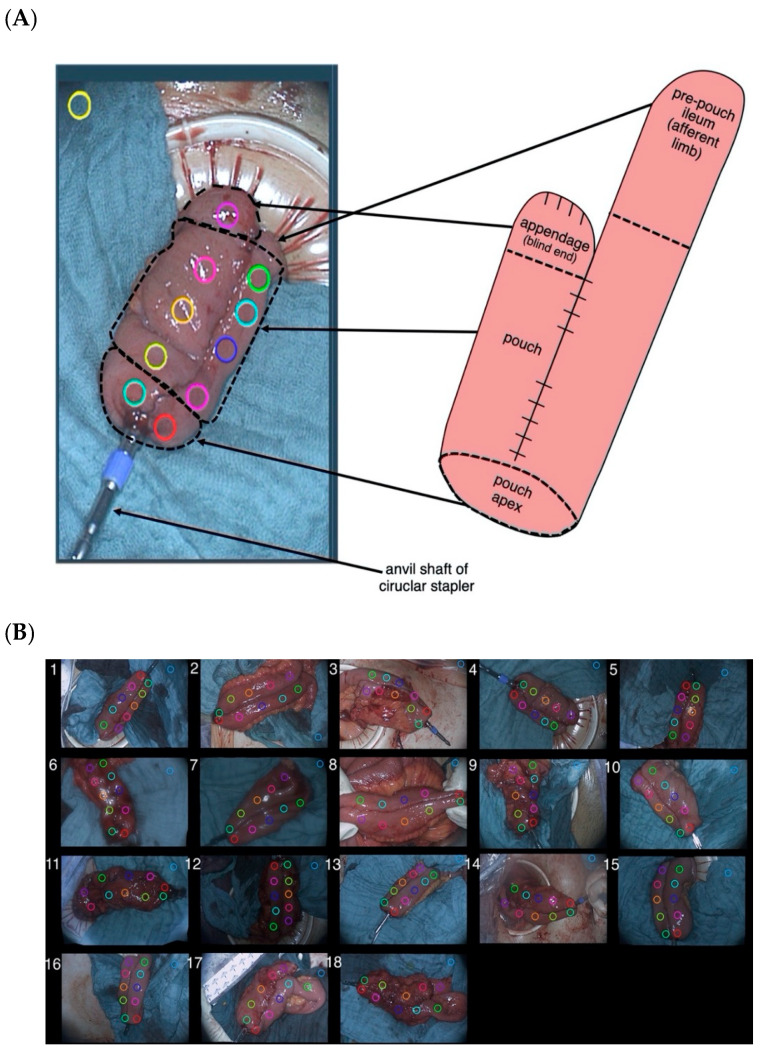
Components of the ileal J-pouch and the respective placement of regions of interest (ROIs). (**A**) For postoperative software-based perfusion assessment of fluorescence intensity over time, 10 regions of interest (ROIs) were manually selected on each pouch visualization in a predefined order: 8 ROIs on the pouch body including the pouch appendage (blind end); two ROIs at the pouch apex, one ROI on the surrounding surgical drape as a negative control to indicate autofluorescence (control ROI). Each ROI was given an individual color to allow a clear allocation of the planned successive assessment (**B**) This was repeated in a standardized manner for all visualization recordings for patients 1 to 18.

Using the Quest Research SoftwareTM, the video recordings underwent a postoperative perfusion assessment by analysing the distribution of fluorescence intensity over time. To achieve this, 10 regions of interest (ROIs) were manually selected on each pouch visualization in a standardized, predefined order: 8 ROIs on the pouch body including the pouch appendage (blind end) as well as two ROIs at the pouch apex (Figure 2A,B). One ROI was placed on the surrounding surgical drape as a negative control to indicate autofluorescence and background noise (control ROI). Each ROI was given an individual color to allow a clear allocation of the planned successive assessment.

By depicting fluorescence intensity over time, a perfusion curve was generated for each ROI, resulting in an individual perfusion graph for each pouch. The shape and characteristics of each perfusion graph were analysed in R statistical software (version number 4.1.2, www.r-project.org, the R foundation, Vienna, Austria; accessed on 1 December 2021). Additionally, a computed pixel-to-pixel analysis of fluorescence intensity over time was conducted for each video recording. In this assessment mode, the software utilized each pixel as an individual ROI, allowing an analysis for maximum ingress and maximum egress and its detailed depiction as a heat map.

### 2.4. Definition of Anastomotic Leak

A standardized definition of AL of the ileal pouch is lacking. Thus, to avoid further ambiguity and improve comparability, in conformity with previous definitions of AL in colorectal surgery [32], we applied the following definition: AL of the pouch is defined as a defect of intestinal wall integrity at the anastomotic sites of the pouch leading to a communication between intra- and extraluminal compartments and an exodus of pouch luminal content (including air, faecal content and abscess formation communicating with the anastomotic site). Diagnosis is made by pouchoscopy and can be radiographically supported by CT, MRI and pouchography with contrast enema. It can occur with or without symptoms of pelvic sepsis. 

### 2.5. Clinical Data and Follow-Up

Detailed clinical follow-up was performed on 30-day postoperative morbidity for all patients, with AL of the pouch as the primary outcome. To screen for this, flexible pouchoscopy was regularly performed in every patient 6–8 weeks after surgery or during the postoperative hospital stay if signs of AL or pelvic sepsis (prolonged postoperative ileus; fever > 38.5 °C; leucocytosis; elevated C-reactive protein) were detected. Additionally, radiologic pouchography with contrast enema was performed 6–8 weeks after surgery.

### 2.6. Primary and Secondary End Points

AL of the pouch as described within 30 postoperative days was defined as the primary end point. Baseline patient characteristics, 30-day postoperative morbidity and the imaging environment were defined as secondary end points, including sex, American Society of Anesthesiologists (ASA) physical status classification, underlying condition, disease category, preoperative immunosuppressive medication, type of surgery, two- or three-stage approach, hand-sewn ileal pouch-anal anastomosis (IPAA), intraoperative change of the anastomotic site, age at operation (years), body mass index (BMI) and the presence of autofluorescence in the imaging environment.

### 2.7. Sample Size

We applied a novel assessment method investigating the correlation of novel perfusion indicators with clinical outcome, discriminating between cases of AL and no AL. No previous data investigating this specific correlation existed prior to study commencement to enable the prediction of a suitable sample size. Based on the results of this study, power analysis for sample size may be possible in future studies, taking into account the prevalence of AL.

## 3. Results

**Figure 3:**
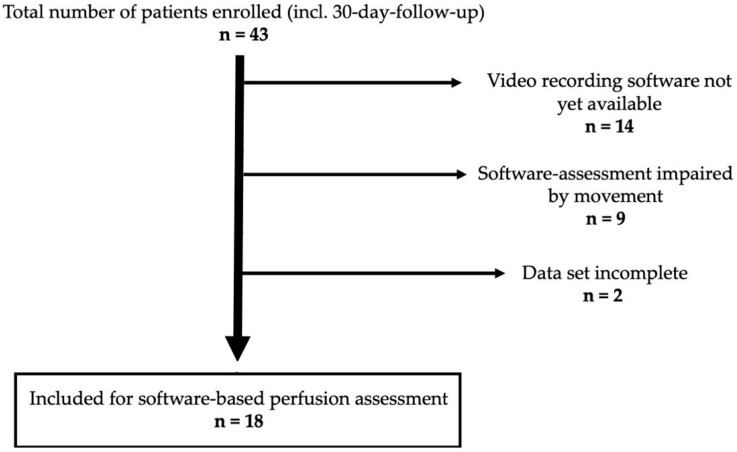
Flow-chart illustrating the number of patients included for software-based perfusion assessment and statistical analysis due to the necessity of a movement-free video recording and strict accrual/exclusion criteria to generate high quality data.

In total, 18 patients underwent ileal J-pouch with intraoperative ICG-NIRF visualization and video recording, follow-up, and statistical and perfusion assessment analysis (Table 1). Reasons for exclusion from the enrolled total of patients that completed clinical follow-up were an initial non-availability of a video recording of the perfusion visualization, impairment by movement and an incomplete data set with regards to intraoperative additional data (Figure 3). Four out of 18 patients developed AL of the IPAA within 30 days (AL group), all of them with grade A leak. No AL occurred at the pouch body or appendage. The other 14 patients did not develop AL of the pouch (non-AL group). Intraoperative ICG-NIRF perfusion visualization was achieved with a sufficient subjective signal strength and time to signal in all cases. There was no intraoperative change of anastomotic site after perfusion visualization.

**Figure 4:**
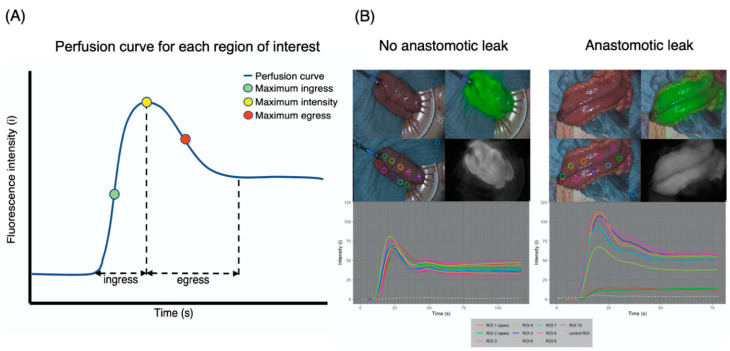
Perfusion graph consisting of perfusion curves for each region of interest and their characteristics. (**A**) The software-based depiction of fluorescence intensity over time per ROI resulted in a perfusion graph for each pouch. Distinctive perfusion curves of the same color were generated for each individually colored ROI, allowing a differentiation between pouch body and pouch apex. The shape of the perfusion curves for non-AL pouches was similar and aligned synchronously with the other ROIs of the pouch, consisting of a fluorescence ingress phase, intensity peak and egress phase (example on the left). In AL pouches, the perfusion curves of ROIs on the pouch apex (ROI 1 and ROI 2) did not show alignment with the other ROIs, suggesting impaired perfusion (example on the right). (**B**) Recurring time points and characteristics of the perfusion curves could be identified. The ingress phase, defined by a positive slope of the perfusion curve, represents the inflow of blood at the ROI. Maximum slope represents the maximum rate of the inflow of blood (maximum ingress). Maximum intensity marks the point at which the fluorescent signal has reached its maximum intensity and marks the beginning of the outflow of blood (i.e., egress phase). Egress represents slope starting at the time of maximum intensity and ends at the time point 5 s after the time of maximum intensity and is calculated according to Formula (1).

The software-based analysis resulted in a depiction of fluorescence intensity measured per pixel (i: intensity) over time in seconds (s) for the ROIs of each pouch, resulting in a rate of intensity change per second (i/s). A distinctive perfusion curve was generated for each ROI, allowing the differentiation between pouch body, pouch apex and pouch appendage. A general shape could be observed in most cases, which was similar and aligned synchronously with the other ROIs of the same pouch. A fluorescence ingress (i.e., inflow) phase was followed by an intensity peak and egress (i.e., outflow) phase (Figure 4A). 

Thus, recurring time points and characteristics of the perfusion curves were identified (Figure 4B): “Ingress begin” marked the beginning of the ingress phase, defined by a positive slope of the perfusion curve, representing the inflow of blood at the ROI. “Maximum slope” of the perfusion curve represented the point at which the inflow of blood (ingress) had reached its maximum rate. “Maximum intensity” represented the point at which the fluorescent signal had reached its maximum intensity and marked the beginning of the outflow of blood (i.e., *egress*). “Maximum intensity + 5 s” represented the time point 5 s after the maximum fluorescence intensity was reached. To calculate the slope for the *egress* phase (*egress*), the intensity value at the time point of maximum intensity (*I_tmax_*) and the intensity value at the time point 5 s after the maximum intensity was reached (*I_tmax+_*_5_) were used (Formula (1)).

(1) $$egress=\frac{I_{tmax+5}-I_{tmax}}{5}$$

Differences in the shapes of the perfusion graphs became evident by preliminary visual comparison. In the AL group, ROIs of the pouch apex (directly adjacent to the IPAA) predominantly showed weaker ingress, peak and egress as well as weaker overall fluorescence intensity when compared to the non-AL pouches (Figure 4B).

**Figure 5:**
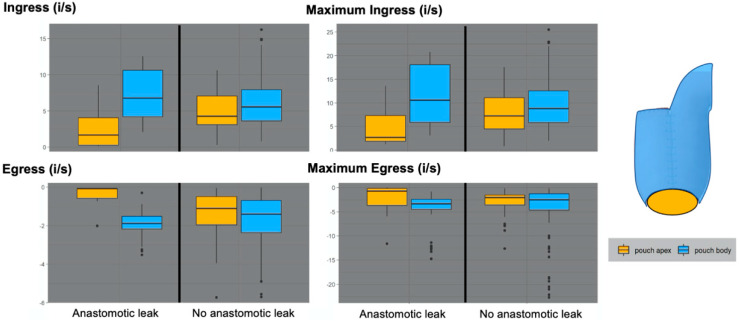
Novel parameters of the perfusion curves correlated with outcome and location on the pouch. The novel characteristics of the perfusion curve identified (ingress, maximum ingress, egress and maximum egress) were correlated with the clinical outcome of each pouch. A differentiation was further made between ROIs on the pouch body and the pouch apex. The ingress of ROIs on the pouch body was similar in both AL- and non-AL cases. In contrast, the ingress of ROIs on the pouch apex was considerably lower in the AL group. The same distribution was evident for maximum ingress, which represents the maximum positive slope of the perfusion curve. The egress of ROIs on the pouch body was similar in AL and non-AL cases, whereas ROIs on the pouch apex displayed considerably lower egress in cases of AL, representing a delayed or impaired outflow of blood. The same distribution was seen for maximum egress. In summary, ROIs on the pouch apex displayed lower ingress, maximum ingress, egress and maximum egress values in cases of AL, while ROIs on the pouch body showed no considerable difference. In non-AL cases, all parameters for both the pouch apex and pouch body had an overlapping range. Conversely, in cases of AL, there was a notable difference between pouch apex and pouch body.

The identified novel characteristics of the perfusion curve (ingress, maximum ingress, egress and maximum egress) were examined in depth for each pouch according to clinical outcome and ROI location on the pouch body or pouch apex (Figure 5). The corresponding numerical values are presented in detail in Table 2.

The ingress of ROIs on the pouch body was similar in both AL (median = 6.8; range = 10.5 {12.6–2.1}; interquartile range = 6.4 {8.0–3.6} i/s) and non-AL cases (median = 5.6; range = 13.3 {14.1–0.8}; interquartile range = 4.3 {8.0–3.6} i/s). In contrast, the ingress of ROIs on the pouch apex was considerably lower in the AL group (median = 1.7; range = 8.5 {8.6–0.1}; interquartile range = 3.8 {4.1–0.3} i/s) than in the non-AL group (median = 4.3; range = 10.3 {10.6–0.3}; interquartile range = 4.0 {7.1–3.1} i/s), indicating an impaired inflow at the apex.

The same distribution was evident for the maximum ingress, which represented the maximum positive slope of the perfusion curve.

Concordantly, the egress of ROIs on the pouch body was similar in both AL (median = −1.9; range = 2.2 {(−0.9)–(−3.1)}; interquartile range = 0.7 {(−1.5)–(−2.2)} i/s) and non-AL cases (median = −1.4; range = 4.8 {0.0–(−4.8)}; interquartile range = 1.7 {(−0.7)–{{−2.4)} i/s). Contrarily, the ROIs on the pouch apex displayed considerably lower egress in cases of AL (median = −0.1; range = 0.7 {0.0–(−0.7)}; interquartile range = 0.5 {(−0.1)–(−0.6)}i/s) than in non-AL cases (median = −1.1; range = 3.9 {0.0–(−4.0)}; {0.0–(−0.7)}; interquartile range = 1.5 {(−0.5)–(−2.0)} i/s), indicating an impaired outflow at the apex. The maximum egress value of ROIs of the pouch body was again similar for both outcomes, while the ROIs of the pouch apex showed lower maximum egress values in cases of AL. 

In summary, ROIs on the pouch apex displayed lower ingress, maximum ingress, egress and maximum egress values in cases of AL, while ROIs on the pouch body showed no considerable difference. In non-AL cases, all parameters for both the pouch apex and pouch body had an overlapping range. Conversely, in cases of AL, there was a notable difference between pouch apex and pouch body. 

**Figure 6:**
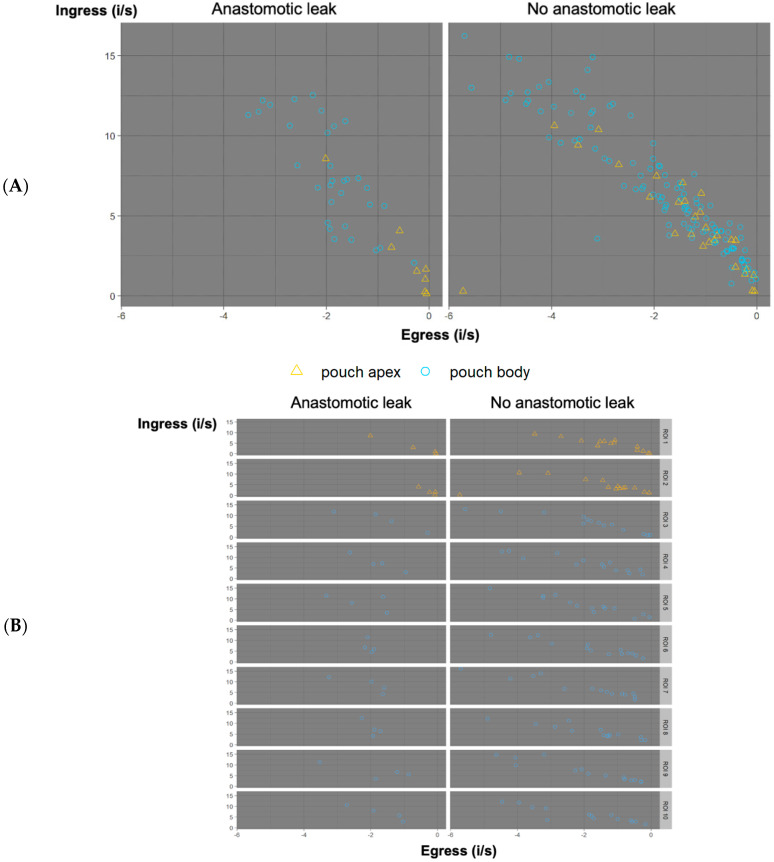
(**A**) Ingress versus egress. Ingress is displayed on the *y*-axis. Egress is displayed on the *x*-axis. ROIs are displayed by outcome as “anastomotic leak” or “no anastomotic leak”. They are further differentiated by location on the pouch body (blue circle) or pouch apex (yellow triangle). Pouches with a regular outcome showed an evenly spread-out distribution of both ROIs of the pouch body and pouch apex. AL pouches showed considerably lower ingress and egress values for ROIs on the pouch apex, while ROIs on the pouch body showed no considerable difference. (**B**) Distribution of ingress and egress per ROI according to clinical outcome. Maximum ingress is displayed on the *y*-axis, egress is displayed on the *x*-axis for each ROI, respectively, sorted by outcome “anastomotic leak” and “no anastomotic leak”. For cases of AL, ROIs on the pouch apex (ROIs 1 and 2) exhibit considerable grouping at low ingress and egress values, while ROIs of the pouch body appear evenly spread out, especially in non-AL cases.

In the analysis of ingress versus egress (Figure 6), pouches with a normal outcome showed an evenly spread-out distribution of ROIs on the pouch body and pouch apex. Pouches with AL showed considerably lower ingress and egress values for ROIs of the pouch at the apex (Figure 6A). This was also the case when comparing the distribution of ingress and egress for each individual ROI location in AL cases with non-AL cases (Figure 6B). In cases of AL, ROIs on the pouch apex (ROIs 1 and 2) exhibited considerable grouping at low ingress and egress values, while ROIs of the pouch body appeared evenly spread out, especially in non-AL cases.

The computed pixel-to-pixel analysis of the video recordings resulted in a parametric map for maximum ingress and maximum egress (Figure 6). The parametric mapping required pixel-for pixel registration and was not possible in recordings with even minimal movement due to bowel movement (patients 4, 6, 8, 10, 16, 17). It was successful in patients 2, 3, 5, 7, 9, 11, 12, 13, 14, 15, and 18.

**Figure 7:**
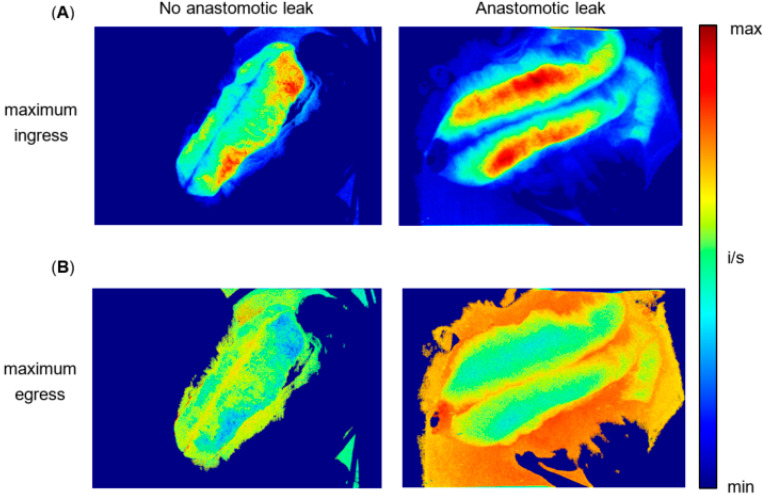
(**A**) Computed parametric pixel-to-pixel map of maximum ingress over time for patient 1 (non-AL) and patient 2 (AL). Each pixel of the video recording was analysed as an ROI of maximum ingress over time, resulting in a parametric map showing the distribution of maximum ingress values across a pouch developing AL. The patient on the left (no anastomotic leak), exhibits evenly distributed values (red, yellow and green) on both pouch apex and pouch body. The patient on the right (anastomotic leak) displays minimal values of maximum ingress (blue) on the pouch apex (adjacent to the IPAA), while the pouch body shows medium (yellow) to maximum values (red). (**B**) Computed parametric pixel-to-pixel map of maximum egress over time for patient 1 (non-AL) and patient 2 (AL). Each pixel of the video recording was analysed as an ROI of maximum egress over time, resulting in a map showing the distribution of maximum egress values across a pouch developing AL. The patient on the left (no anastomotic leak) displays medium to high values on both pouch body and apex. The patient on the right (anastomotic leak) exhibits an area of minimal values on the distal pouch apex, while the pouch body also displays medium to high values.

In the resulting parametric map, each pixel of the recording was analysed as an ROI for maximum ingress and maximum egress, respectively, resulting in an objective high-resolution map of each pouch. For clarity and to enable direct comparison, we have shown the parametric mapping for patient 1 (non-AL) and patient 2 (AL) from Figure 3 (Figure 7).

In maximum ingress mapping (Figure 7A), patient 1 (non-AL), exhibited evenly distributed values (red, yellow and green) on both pouch apex and pouch body. Patient 2 (AL) displayed minimal values of maximum ingress (blue) om the pouch apex (adjacent to the IPAA), while the pouch body showed medium (yellow) to maximum values (red).

In maximum egress mapping (Figure 7B), patient 1 displayed medium to high values on both pouch body and apex. Patient 2 (AL) exhibited an area of minimal maximum egress values on the pouch distal apex, while the pouch body also displayed medium to high values.

## 4. Discussion

The study presented here provides a novel, systematic methodology (with standardized measuring time points, dosage and imaging environment) to quantitatively assess anastomotic perfusion with ICG-NIRF and clinical outcome in ileal J-pouch surgery. Furthermore, this study describes a novel multi-step software-based assessment with objective indicators of pouch perfusion and areas of impaired perfusion, which are described for the first time in the medical literature.

In studies investigating anastomotic perfusion with ICG-NIRF, the ICG dosage varies considerably, while the standardization of measuring time points, surrounding light, camera distance to target, device settings (auto-visualization or signal amplification modes) are rarely ever mentioned [23,24,33]. However, these are factors that may influence the fluorescence signal detected and are thus potential confounders to a quantitative, objective and comparable perfusion assessment. In the reported studies investigating ileal pouch perfusion with ICG-NIRF, the study settings were not standardized accordingly [34,35,36]. Spinelli et al. performed an interesting pioneering study on the ileal pouch; however, the intraoperative visualization, the devices used, and follow-up were not quantitative or comparable [37]. The methodology presented in our study strictly standardized and recorded the imaging environment (dosage, timing, camera distance to target, surrounding light, device settings, patient vital signs), providing comparability. As we have recently shown, even with such a systematic imaging approach, the subjective interpretation of the fluorescence signal alone may not be sufficient to indicate impaired perfusion in cases of AL [38].

However, the combination of this systematic imaging approach combined with a novel software-based perfusion assessment resulted in quantifiable results. A graphic depiction of fluorescence intensity over time for defined ROIs resulted in perfusion graphs, which in turn were composed of perfusion curves following a reproducible pattern. So far, only a few clinical feasibility studies mention perfusion-time curves; however, none investigate the ileal J-pouch in a standardized manner or examine ingress and egress in detail [39,40].

For the first time in the medical literature, novel parameters of pouch perfusion were identified by examining the perfusion curves of the pouch apex and pouch body according to clinical outcome with regards to AL. The possible significance of the slope of the perfusion curve for AL in software-based assessment has previously been described, while ingress and egress have not [29]. Maximum intensity has been described to be influenced by factors such as blood pressure [41]. It is also likely to be affected by the imaging environment (e.g., surrounding visible light), which was strictly controlled in our study. Our results suggest that absolute maximum intensity values are less representative than the detailed changes in intensity over time.

The applied methodology resulted in perfusion graphs for each pouch consisting of repeatable, similar perfusion curves per ROI. Non-imaging related baseline patient and intraoperative factors did not show a statistical difference with regards to AL of the pouch, verifying high internal validity. The differences in the perfusion graphs of the AL and non-AL group are mechanistically comprehensible, as are the differences in ROIs of the pouch apex and pouch body. Impaired perfusion at the pouch apex is likely to contribute to AL of the IPAA. Interestingly, when ingress, maximum ingress, egress and maximum egress for all pouches (regardless of ROI location) were compared for regular outcome and AL, there was no considerable difference for cases of AL. However, when the differentiation was made between ROIs located on the pouch body and the pouch apex (Figure 4), there was a difference in all of the respective values. This suggests that in order to evaluate anastomotic perfusion and predict clinical outcome, it is not sufficient to collectively assess a (large) surgical area of interest, but rather that an accurate differentiation and resolution are needed. It points towards the possibility that micro-perfusion is highly relevant to anastomotic perfusion. Micro-perfusion cannot likely be assessed by examining static intraoperative fluorescence images and overlays. Rather, it needs an objective assessment with a high resolution (i.e., distinctive ROIs) and a standardized visualization methodology—as applied in our study. While the findings are promising, they need to be assessed for statistical significance with a larger sample size in the future.

The novel, dynamic parameters of perfusion identified here represent the rate of inflow and outflow of blood at a region of surgical interest and are mechanistically comprehensible, enabling us to detect impaired perfusion not only due to reduced blood supply but also due to venous congestion. This is a feature that the widespread subjective real-time interpretation of ICG-NIRF has not been able to deliver. The relevance of this study is emphasized by the urgent need for objective and comparable intraoperative perfusion assessment and follow-up in ileal pouch surgery, which can only be achieved by systematic standardization of ICG-NIRF visualization and quantitative, software-based protocols such as those proposed here.

Our study included a limited number of patients and can be improved, which we would like to address in the future by increasing the number of patients included, introducing randomization and blinded assessment to investigate statistical significance. The three time points of visualization had different degrees of relevance for predicting AL of the pouch. Time point T1 (ileal segment before pouch construction) was designed to indicate baseline perfusion and to exclude an overall perfusion deficit, which was not the case in any patient. Time point T2 (J-pouch after fashioning) allowed visualization and assessment of the whole J-pouch, which is why it was given the highest priority for assessing pouch perfusion. Time point T3 (intraluminal visualization of ileal pouch-anal anastomosis after circular stapling) had a high theoretical relevance to assess anastomotic perfusion locally at the IPAA. However, the intraluminal measurement was technically difficult to standardise in terms of the distance from the camera to the imaging site and reducing movement. Software-based assessment was not yet possible. This needs to be addressed in the future, when flexible ICG-NIRF endoscopes will offer additional intraluminal visualization possibilities. 

The parametric pixel-to-pixel mapping holds great promise for intraoperative real-time use, as it is highly accurate and removes further subjectivity and bias from the perfusion assessment (no manual ROI placement is required, as every pixel represents an ROI). We have recently shown its feasibility for perfusion assessment in gracilis muscle interposition [42]. However, to implement its widespread use, further optimisation of intraoperative visualization, recordings and movement-correction software are needed, as our results reflect: for several recordings, the pixel-to-pixel analysis was not successful due to movement in the recording caused primarily by intestinal peristalsis and secondarily by aortic pulse (patients 14, 4, 6, 8, 10, 16, 17). This needs to be addressed in upcoming trials by adding improved motion-correction software.

Requiring less than one minute of computing time, the software-based perfusion assessment could have technically been performed intraoperatively in real-time; however, to increase patient safety, we decided to perform a retrospective analysis first to prove its feasibility before altering intraoperative decision making. To further investigate whether the novel parameters of perfusion identified here are indeed objective indicators of perfusion, larger, randomized and blinded trials are needed to investigate statistical significance.

## 5. Conclusions

This study provides the feasibility of a methodology to objectively assess anastomotic perfusion with ICG-NIRF and clinical outcome in pouch surgery. By analysing and depicting fluorescence intensity over time dynamically, perfusion graphs could be characterized in detail and correlated with clinical outcome. Novel parameters of pouch perfusion such as ingress, maximum ingress, egress and maximum egress could be identified and confirmed by parametric pixel-to-pixel mapping. They have never been described before and have the potential to improve the objectivity and quantifiability of intraoperative perfusion assessment in the future in real-time, not only for pouch surgery, but also for intestinal surgery in general.

## Figures and Tables

**Table 1:** Baseline patient data.

Characteristic		All Patients (*n* = 18)	No Anastomotic Leak (*n* = 14)	Anastomotic Leak
Sex	male	8	6	2
	female	10	8	2
ASA physical status	I	1	1	0
	II	14	11	3
	III	3	2	1
Underlying condition	ulcerative colitis	17	14	3
	Crohn’s disease	0	0	0
	indeterminate colitis	1	0	1
Disease category	malignant	1	1	0
	medically refractory	17	13	4
Preoperative prednisolone	yes	0	0	0
	no	18	14	4
Additional immunosuppressive medication	yes	5	2	3
	no	13	12	1
Type of surgery	laparoscopic	14	11	3
	open	4	3	1
Two- or three-stage approach	Two-stage	3	1	2
	Three-stage	15	13	2
Hand-sewn IPAA	yes	4	4	0
	no	14	10	4
Intraoperative change of anastomotic site	yes	0	0	0
	no	18	14	4
Age at operation (years)	mean	35.8	34.4	40.7
	median	34.4	32.0	35.9
	range	19.9–57.0	19.9–57.0	34.8–56.0
BMI (kg/m^2^)	mean	24.9	25.0	24.7
	median	24.7	24.7	23.7
	range	18.2–34.6	18.0–34.6	18.2–33.1

Abbreviations: *n* = number; AL = anastomotic leak; ASA status = American Society of Anesthesiologists (ASA) physical status classification; IPAA = ileal pouch-anal anastomosis; IQR = interquartile range; BMI = body mass index; kg = kilograms; m^2^ = square metres.

**Table 2:** Median, range and interquartile range values for novel perfusion indicators by outcome and location on pouch body or apex.

	No Anastomotic Leak (*n* = 14)		Anastomotic Leak (*n* = 4)	
	Pouch Body	Pouch Apex	Pouch Body	Pouch Apex
**Ingress (i/s)**				
**Median**	5.6	4.3	6.8	1.7
**Range** **{maximum–minimum}**	13.3 {14.1–0.8}	10.3 {10.6–0.3}	10.5 {12.6–2.1}	8.5 {8.6–0.1}
**IQR** **{P_75%_–P_25%_}**	4.3 {8.0–3.6}	4.0 {7.1–3.1}	6.4 {8.0–3.6}	3.8 {4.1–0.3}
**Egress (i/s)**				
**Median**	−1.4	−1.1	−1.9	−0.1
**Range** **{maximum–minimum}**	4.8 {0.0–(−4.8)}	3.9 {0.0–(−4.0)}	2.2 {(−0.9)–(−3.1)}	0.7 {0.0–(−0.7)}
**IQR** **{P_75%_–P_25%_}**	1.7 {(−0.7)–(−2.4)}	1.5 {(−0.5)–(−2.0)}	0.7 {(−1.5)–(−2.2)}	0.5 {(−0.1)–(−0.6)}
**Maximum ingress (i/s)**				
**Median**	8.8	7.2	10.6	2.7
**Range** **{maximum–minimum}**	20.1 {22.0–2.0}	16.8 {17.6–0.8}	17.7 {20.8–3.1}	12.4 {13.6–1.3}
**IQR** **{P_75%_–P_25%_}**	6.7 {12.6–5.8}	6.6 {11.1– 4.5}	12.2 {18.1–5.9}	5.5 {7.3–1.8}
**Maximum egress (i/s)**				
**Median**	−2.5	−2.1	−3.4	−0.7
**Range** **{maximum–minimum}**	7.2 {(−0.1)–(−7.3)}	5.9 {(−0.2)–(−6.1)}	4.7 {(−0.8)–(−5.5)}	5.9 {0.0–(−5.9)}
**IQR** **{P_75%_–P_25%_}**	3.4 {(−1.3)–(−4.6)}	2.0 {(−1.5)–(−3.6)}	2.1 {(−2.4)–(−4.5)}	3.5 {(−0.2)–(−3.7)}

Abbreviations: *n* = number; i = intensity value measured per pixel; s = second, i/s = rate of intensity change per second; IQR = interquartile range; P_75%_ = 75th percentile; P_25%_ = 25th percentile.

## Data Availability

Not applicable.
